# Two Concurrent Outbreaks of *Listeria monocytogenes* Infections Linked to Packaged Salads, United States, 2014–2022

**DOI:** 10.3201/eid3112.250989

**Published:** 2025-12

**Authors:** Alexandra Palacios, Michael Vasser, Asma Madad, Stranjae Ivory, Lauren Edwards, Danielle Donovan, Tiffany Greenlee, Tim Muruvanda, Joseph Baugher, Kurt Nolte, Annemarie Buchholz, Karen Blickenstaff, Mabel Low, Grace Pederson, Jasmine Huffman, Carrie Crabtree, Colby Brown, Meghan Hamel, Rima Kandar, Erin Szidonya, Hannah Caird, Katie Cibulskas, Brandi Taylor, Jodi Taylor, Yamir Rosa, Laura Gieraltowski, Amanda Conrad

**Affiliations:** Centers for Disease Control and Prevention, Atlanta, Georgia, USA (A. Palacios, M. Vasser, M. Low, G. Pederson, J. Huffman, L. Gieraltowski, A. Conrad); US Food and Drug Administration, College Park, Maryland, USA (A. Madad, S. Ivory, T. Greenlee, T. Muruvanda, J. Baugher, K. Nolte, A. Buchholz, K. Blickenstaff); Michigan Department of Agriculture and Rural Development, Lansing, Michigan, USA (L. Edwards); Michigan Department of Health and Human Services, Lansing (D. Donovan): Georgia Department of Agriculture, Atlanta (C. Crabtree, C. Brown); Public Health Agency of Canada, Ottawa, Ontario, Canada (M. Hamel, R. Kandar); National Microbiology Laboratory, Winnipeg, Manitoba, Canada (E. Szidonya); British Columbia Centre for Disease Control, Vancouver, British Columbia, Canada (H. Caird); Ohio Department of Health, Columbus, Ohio, USA (K. Cibulskas, B. Taylor); Ohio Department of Agriculture, Reynoldsburg, Ohio, USA (J. Taylor, Y. Rosa)

**Keywords:** Listeria, Listeria monocytogenes, bacteria, food safety, outbreak, listeriosis, romaine, packaged salads, leafy greens, foodborne illness outbreaks, iceberg, United States

## Abstract

We describe 2 genetically unrelated outbreaks of *Listeria monocytogenes* infections (outbreak A and outbreak B) linked to packaged salads from 2 different firms that were investigated simultaneously in 2021. Combined, the outbreaks caused 30 illnesses, 27 hospitalizations, and 4 deaths over 8 years. Those investigations led to recalls of product from 2 different firms and highlight how *L. monocytogenes* contamination can persist for long periods and cause illnesses over many years. Outbreak A was investigated 3 times with illnesses occurring over 8 years, whereas illnesses in outbreak B occurred over 5 years. Both outbreaks illustrate the importance of routine and epidemiologically directed sampling by state partners, without which these outbreaks likely would have gone unsolved. The outbreaks were the second and third multistate outbreaks of listeriosis linked to packaged salads, providing further documentation of the potential for *L. monocytogenes* infections from consumption of contaminated packaged salads.

Before 2015, listeriosis outbreaks linked to produce items in the United States were linked to melons, sprouts, and celery. At least 1 sporadic listeriosis case in 2014 and 1 binational listeriosis outbreak in 2015–2016 were linked to packaged salads (*1*). We describe an unusual situation of 2 genetically unrelated outbreaks of *Listeria monocytogenes* infection in the United States (outbreak A and outbreak B), investigated simultaneously in late 2021, that were linked to packaged salads from 2 different firms. Of those outbreaks, 1 also included genetically related cases in Canada. The Centers for Disease Control and Prevention (CDC) and Public Health Agency of Canada (PHAC) regularly exchange information when an outbreak has potential to span national borders.

*Listeria* can survive in production facilities or environments for long periods. Listeriosis outbreak investigations often span multiple years before a food vehicle is identified because of harborage in a production facility or persistent contamination levels in the environment or product, resulting in seemingly sporadic illnesses over long periods (*2*,*3*). Evidence of contamination in California watersheds connected to a major leafy green production region was described in a 2014 study conducted by the US Department of Agriculture (USDA) (*4*) (hereafter referred to as the USDA study), in which investigators found that *L. monocytogenes* was prevalent in 43% of all environmental samples. When water isolates from that study are related to clinical isolates by whole-genome sequencing (WGS), that link gives investigators a clue that leafy greens or other produce grown in that region could be a source of illness.

In listeriosis outbreaks, epidemiologic information is often limited because of relatively low case numbers, long incubation periods, and severity of illness. Patients might be older, have poor food recall, or be too sick to provide food exposure information. Listeriosis outbreaks can take longer to solve than foodborne outbreaks caused by other pathogens and might rely on product or environmental sampling (*5*,*6*). This activity was reviewed by CDC, deemed not research, and conducted consistent with applicable federal law and CDC policy (e.g., 45 C.F.R. part 46.102(l)(2), 21 C.F.R. part 56; 42 U.S.C. §241(d); 5 U.S.C. §552a; 44 U.S.C. §3501 et seq.).

## Methods

### Case Definition

An outbreak A case was defined as invasive listeriosis in a patient from whom an isolate was collected during August 16, 2014–January 15, 2022, and related to the outbreak A strain within 0–21 allele differences by whole-genome multilocus sequence typing (wgMLST). An outbreak B case was defined as invasive listeriosis in a patient whose isolate was collected during July 26, 2016–October 19, 2021, and related to the outbreak B strain within 0–7 allele differences by wgMLST.

### Epidemiologic Data

The *Listeria* Initiative is a national surveillance system coordinated by CDC that collects clinical, demographic, and food exposure data for all listeriosis cases (*7*). State and local health departments interview patients or surrogates about foods eaten in the 28 days before their illness. CDC conducts case–case analyses using SAS version 9.4 (SAS Institute Inc., https://www.sas.com) to compare foods eaten by outbreak patients to foods eaten by sporadic listeriosis patients not associated with an outbreak from the same states. Supplemental questionnaires are deployed to collect more specific product information. Investigators also request records from stores reported by patients for a comprehensive purchase history. PHAC uses similar methods to investigate listeriosis outbreaks (*6*).

### Laboratory Methods

PulseNet USA, CDC’s national molecular subtyping network for enteric disease surveillance, detects clusters of *L. monocytogenes* when 3 clinical isolates related by 0–25 allele differences by wgMLST occur within 120 days (*8*). For strains with genetic diversity, CDC might narrow the allele range to <10 (*9*). Sequencing results and analysis were performed using BioNumerics version 7.6.3 (bioMérieux, https://www.biomerieux.com). All sequenced isolates are uploaded to the National Center for Biotechnology Information (NCBI) Pathogen Detection pipeline (https://www.ncbi.nlm.nih.gov/pathogens). Before the use of wgMLST, pulsed-field gel electrophoresis was performed on isolates uploaded during 2014–2018. Canada uses similar laboratory methods to detect clusters but with narrower criteria (*6*).

Samples of leafy green products, water, or environmental swabs were collected using standard practices and analyzed them for *L. monocytogenes* using the US Food and Drug Administration (FDA) Bacteriological Analytical Manual (https://www.fda.gov/food/laboratory-methods-food/bacteriological-analytical-manual-bam). FDA and firm isolates underwent WGS analysis using the CFSAN SNP Pipeline (*10*).

### Sampling

State and local public health partners conduct routine surveillance sampling and epidemiologically directed sampling. Routine sampling is done at regular intervals, independently from outbreak investigations. In Michigan Department of Agriculture and Rural Development (MDARD) routine sampling, inspectional and laboratory staff collect samples for microbiological testing. During a sampling event, inspectors obtain samples of high-risk or empirically driven commodities to test for *Salmonella*, *Listeria*, and Shiga toxin–producing *Escherichia coli*. The Georgia Department of Agriculture (GDA) Retail Risk-Based Surveillance Program uses a set schedule for retail inspectors to obtain samples from retail locations for laboratory testing during routine inspections.

Epidemiologically directed sampling is conducted in multistate outbreaks when investigators suspect a food vehicle as the source of an outbreak but require additional laboratory evidence to link illnesses to a product. Officials visit retail locations reported by patients to sample suspected foods identified through interviews or collected records.

### Traceback

The FDA conducts traceback in multistate outbreaks using previously described methods (*11*) to determine whether suspected food products come from a common source. FDA conducts full-scope Preventive Controls for Human Food inspections at processing facilities of interest, which require facilities to have food safety plans, including hazard analysis and risk-based preventive controls. Inspections also include collecting traceability records and samples.

## Results

### Outbreak A

#### 2019–2020

On January 28, 2019, PulseNet detected a cluster of *L. monocytogenes* clinical isolates related within 0–10 allele differences by wgMLST. Epidemiologic information was insufficient to identify a source, and the investigation was closed on April 12, 2019; a total of 5 cases were identified in 5 states (Iowa, Maryland, Ohio, Pennsylvania, and Texas).

On December 10, 2019, after PulseNet identified 4 additional cases related within 0–10 allele differences by wgMLST to the previous cases, CDC opened a second investigation. PHAC identified 2 cases highly related to the outbreak strain in 2 Canada provinces. One Canada patient reported consuming brand X packaged coleslaw. Packaged salad exposure information was unavailable for the second case. Grocery store locations were shared with the Canadian Food Inspection Agency, which confirmed that brand X packaged coleslaw, sourced from a US processing facility, was available for purchase before the patient’s illness onset.

Packaged salads were the most reported exposure among US patients; a leafy greens supplemental questionnaire was deployed. All 5 patients reported consuming packaged salads. Among 3 patients who recalled the brand names of packaged salad, 2 patients reported brands of packaged salad produced by firm X and 1 reported packaged salad from retail chain K. No patients reported consuming coleslaw. Compared with sporadic cases, a case–case analysis showed outbreak case-patients were more likely to have consumed packaged salads before illness (odds ratio [OR] 24.14 [95% CI 4.33–∞]).

The Ohio Department of Agriculture conducted epidemiologically directed sampling and collected 31 samples of packaged salads at retail locations reported by patients. Sampling did not yield *L. monocytogenes*, but *L. welshimeri* was isolated from retail chain K–brand packaged coleslaw produced by firm X’s Ohio facility. The presence of *Listeria* bacteria suggested that conditions were also suitable for survival and growth of *L. monocytogenes* (*12*). On the basis of those sampling results and patient geographic distribution, FDA collected records and samples from firm X facilities in North Carolina and Ohio in January 2020. No food safety observations of concern were noted during the Ohio facility inspection. The North Carolina facility inspection noted issues related to identifying preventive controls, monitoring sanitation controls, written procedures for monitoring process controls, and inadequate design, cleaning, and maintenance of equipment and utensils. *L. monocytogenes* was not detected in samples. One coleslaw sample collected at firm X’s Ohio facility yielded *L. welshimeri*.

Epidemiologic data suggested that packaged salads produced by firm X could have been the source of illness but were not confirmed as the cause. Investigators closed the second investigation on February 10, 2020, after no additional illnesses were identified. The outbreak yielded a total of 11 cases from 8 US states (Iowa, Maryland, North Carolina, Ohio, Oregon, Pennsylvania, Texas, and Wisconsin) and 2 Canada provinces with specimen collection dates during August 16, 2014–November 20, 2019.

#### 2021

On October 18, 2021, GDA conducted routine sampling unrelated to the outbreak investigation and identified *L. monocytogenes* in brand X packaged garden salad produced at firm X’s North Carolina facility. Firm X voluntarily recalled packaged garden salad products on October 29, 2021 (*13*).

On November 1, 2021, CDC opened the investigation a third time when PulseNet identified 7 additional cases. Interviews and record review showed that 10 of 11 patients with exposure information (91%) reported eating packaged salads. Of 4 patients who had brand information, 3 reported consuming brand X and 1 reported consuming brand Y. On December 3, 2021, sequencing showed the brand X packaged garden salad isolate collected by GDA was highly related to the outbreak strain within 0–18 allele differences by wgMLST. To determine whether contaminated product was still on the market, MDARD conducted epidemiologically directed sampling at retail locations, including a store where a patient purchased packaged salad, and collected 37 samples. On December 21, 2021, MDARD identified *L. monocytogenes* in a bag of brand X packaged iceberg lettuce produced in firm X’s Arizona facility. The isolate was highly related to the outbreak strain within 0–18 allele differences by wgMLST.

**Figure 1:**
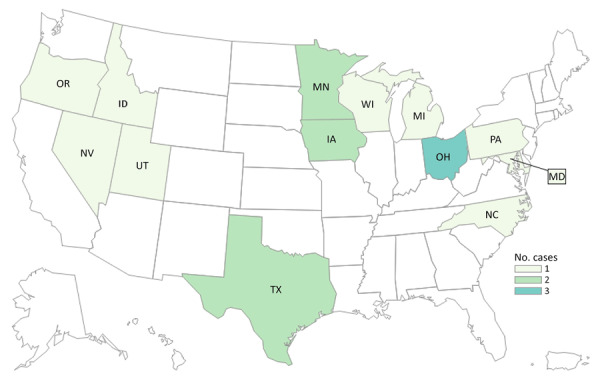
Cases of *Listeria monocytogenes* infection in outbreak A (n = 18), by state of residence, in study of 2 concurrent outbreaks linked to packaged salads, United States, 2014–2022. Colors indicate number of cases per state.

**Figure 2:**
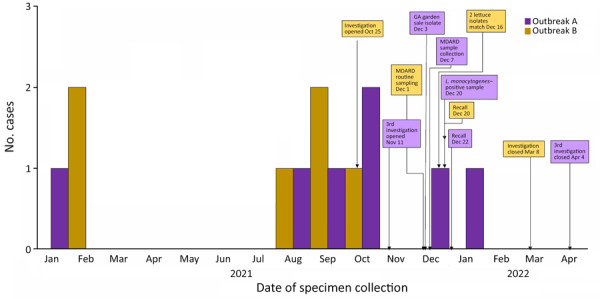
Epidemiologic curve and timeline for 2 outbreaks of *Listeria monocytogenes* infections linked to packaged salads, United States, 2021–2022 (n = 13 cases). Epidemiologic curve only shows US cases from outbreak A and B during 2021–2022. Both cases from Canada occurred in 2019. MDARD, Michigan Department of Agriculture and Rural Development.

By investigation closure, the outbreak included 20 cases from 13 states (Iowa, Idaho, Maryland, Michigan, Minnesota, North Carolina, Nevada, Ohio, Oregon, Pennsylvania, Texas, Utah, and Wisconsin) (Figure 1) and 2 Canadian provinces. Specimens were collected during August 16, 2014–January 15, 2022 (Figure 2). Age range was 50–94 (median 76) years; 17/20 case-patients were women. Seventeen patients were hospitalized, and 3 died. No illnesses were associated with pregnancy.

#### Traceback and Inspection

Because of limited exposure information, FDA did not conduct formal traceback analysis. Products associated with any single firm X facility could not account for the geographic distribution of patients in the outbreak. On the basis of additional illnesses, *L. monocytogenes*–positive samples collected by GDA and MDARD, and other investigational information, FDA ultimately conducted inspections at firm X facilities in Arizona, California, North Carolina, and Ohio. Firm X also initiated an internal investigation that included collecting product and environmental samples. FDA inspections at the 4 firm X facilities involved collection of 60 product and environmental samples. FDA sampling did not yield *L. monocytogenes*, but *L. welshimeri* and *L. innocua* were detected in 1 environmental sample and *L. innocua* was detected in 1 finished product sample, both collected from the North Carolina facility.

#### Control Measures

On December 21, 2021, after the sample collected by MDARD yielded *L. monocytogenes*, firm X halted production at their North Carolina and Arizona facilities. On December 22, 2021, firm X voluntarily recalled all brand X and private-label packaged salads processed at the 2 facilities (*14*). CDC and FDA published announcements on December 22, 2021, warning consumers not to eat recalled products (*15*,*16*).

Firm X conducted a root-cause analysis to determine the source of the lettuce contamination and, in particular, to determine how 2 bags of packaged salads, produced 7 weeks apart in separate facilities, could contain the same strain of *L. monocytogenes*. That analysis was further complicated because iceberg lettuce in the salads was sourced from different regions. Firm X identified a common harvest rig used to harvest the iceberg lettuce in both packaged products collected by GDA and MDARD; environmental swab specimens from the harvest rig yielded the outbreak strain. Firm X’s root-cause analysis concluded the contaminated harvest rig harbored the outbreak strain and the harvest process enabled introduction into the supply chain (N. Dyenson, firm X, pers. comm., email, 2023 Apr 21). After the December 2021 recall, firm X conducted product sampling, which yielded the outbreak strain. FDA analysis verified genetic relatedness. On January 7, 2022, after identifying the outbreak strain on the harvest rig, firm X conducted an additional voluntary recall to include all brand X and private-label packaged salads containing iceberg lettuce processed at their Ohio and California facilities (*17*).

Firm X permanently decommissioned the harvest rig, developed enhanced sanitation protocols, and implemented measures to reduce contamination routes. Firm X also implemented WGS as a tool for early surveillance, integrated microbiological surveillance of incoming raw material, and proactively engaged industry colleagues. Firm X played a collaborative role with FDA and CDC during the outbreak investigation by providing regular updates related to their own investigation and sharing isolates and sequencing data.

#### Additional Laboratory Findings

Several *L. monocytogenes* water-sediment isolates from the USDA study were uploaded to the NCBI Pathogen Detection pipeline in 2020 and 2021 (*4*). Of 635 *L. monocytogenes* isolates identified, 72 were related within 35 alleles by wgMLST to outbreak A.

### Outbreak B

#### 2021

On October 22, 2021, PulseNet identified a cluster of 10 clinical isolates related within 1–6 allele differences by wgMLST. Investigators reviewed data in NCBI and found that the clinical isolates were in the same phylogenetic tree as water-sediment isolates collected as part of the USDA study (*4*). That finding led investigators to suspect a produce item as the source of illnesses given the foods grown in the Salinas Valley of California.

CDC conducted a case–case analysis and found exposure to carrots and exposure to herbs were statistically significant (OR 10.78 [95% CI 1.95–∞] for carrots, OR 14.71 [95% CI 1.65–179.39] for herbs). Of 5 patients with information, all reported exposure to carrots and 3 reported exposure to herbs. Packaged salad consumption was reported by 4 of 5 patients and was not significantly higher than the rate of consumption among sporadic cases. No common types or brands were reported. CDC continued to monitor for additional illnesses.

On December 1, 2021, MDARD collected 45 routine retail samples that included packaged salad products. On December 16, 2021, MDARD notified CDC of isolates that were related to the outbreak strain within 0–7 allele differences by wgMLST. The sample was from brand Z packaged romaine and butter lettuce salad mix sourced from the Salinas Valley and produced by firm Z. At the time, only 1 patient reported consuming brand Z packaged iceberg lettuce. Two patients were reinterviewed using a supplemental questionnaire and their purchase records were collected.

**Figure 3:**
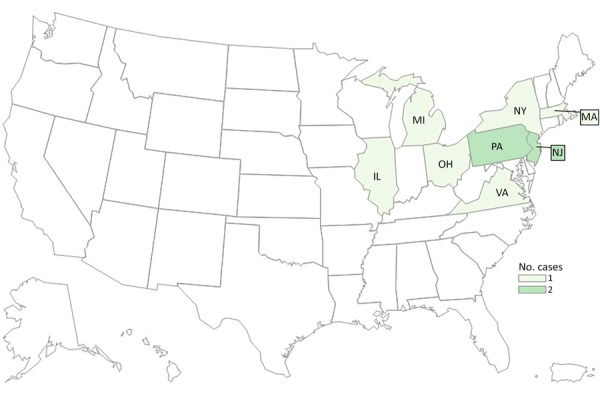
Cases of *Listeria monocytogenes* infection in outbreak B (n = 10), by state of residence, in study of 2 concurrent outbreaks linked to packaged salads, United States, 2014–2022. Colors indicate number of cases per state.

By investigation closure, the outbreak included 10 cases from 8 states (Illinois, Massachusetts, Michigan, New Jersey, New York, Ohio, Pennsylvania, and Virginia) (Figure 3) and 2 food samples with isolates related within 0–7 allele differences by wgMLST. Specimen collection dates were July 16, 2016–October 19, 2021 (Figure 2). Patient age range was 44–95 (median 80) years; 6 of 10 case-patients were women. All 10 patients were hospitalized, and 1 patient died. No illnesses were associated with pregnancy. Four of five patients reported consuming packaged salads; 4 included iceberg and 3 included romaine. Two patients reported consuming brand Z products: 1 reported packaged iceberg lettuce and the other a salad kit containing romaine and baby spinach. No genetically related illnesses were identified in Canada.

#### Traceback and Inspection

In December 2021, FDA initiated a traceback investigation. Because of limited exposure information, only the positive sample collected by MDARD was included in the traceback analysis. On the basis of harvest date information associated with the production code of the positive sample, 3 growers and their 4 respective fields were identified. Because of comingling at firm Z’s Illinois facility, products from each of those fields were used to manufacture the positive sample collected by MDARD.

FDA conducted an environmental investigation at firm Z’s Illinois facility focused on environmental swabbing and review of sanitation practices, procedures, records, and corrective actions. During the inspection, 100 swabs were collected; all were negative for *Listeria* spp*.*

#### Control Measures

Upon notice of MDARD’s positive sample and the related outbreak, firm Z halted production and initiated a complete sanitation review at their Illinois facility. On December 20, 2021, firm Z voluntarily recalled certain varieties of its branded and private-label salad products produced at the Illinois facility (*18*). Recalled product was sent to distributors and wholesalers in the midwestern and northeastern United States and 2 Canada provinces. Product distribution aligned with geographic location of patients in the outbreak. Firm Z’s root cause investigation and environmental monitoring did not identify the source of contamination but informed corrective actions.

CDC and FDA published communications on December 22, 2021, warning consumers not to eat recalled products (*19*). CDC closed the investigation on March 8, 2022, after no new illnesses were identified.

#### Additional Laboratory Findings

Isolates in outbreak B are a rare *L. monocytogenes* sequence type (ST), 639, and exhibit a propensity for water (*20*). The strain was previously found in watersheds from the Salinas Valley (*4*). ST639 was not previously implicated in outbreaks where a source of illness was identified. Of the 4 fields identified in the traceback investigation, 1 was located in the same county where the strain was identified in California watersheds.

## Discussion

Outbreaks A and B, both multistate outbreaks of listeriosis linked to packaged salads, are unique because they were investigated concurrently and highlight how illnesses can occur over multiple years because *L. monocytogenes* contamination can persist for long periods in the natural environment, on harvest equipment, and in the production environment. Outbreak A was investigated 3 times and illnesses occurred over 8 years, whereas illnesses in outbreak B occurred over 5 years. Both outbreaks illustrate the importance of routine and epidemiologically directed sampling by state partners, without which these outbreaks likely would have gone unsolved. Because *Listeria* can form biofilms and survive for extended periods in production facilities, contamination often occurs in the production environment (*12*). Listeriosis outbreak investigations do not commonly identify a root cause of contamination outside of a production environment, such as a specific farm or growing field. The steps firm X took to identify the exact harvest equipment contaminated with the outbreak A strain is a powerful example of data points companies can use to identify points of persistent contamination. Conversely, we speculate that outbreak B was likely caused by environmental contamination at a single production facility. Both outbreaks highlight the ongoing potential for *L. monocytogenes* infections as a result of consuming contaminated packaged salads, especially for high-risk consumers.

Water-sediment isolates collected in the USDA study (*4*) demonstrate the persistence of *Listeria* within an environment where produce is grown (*14*). The genetic relatedness of clinical isolates in the 2 outbreak strains and the California water-sediment isolates provide evidence that *Listeria* could be persistent in growing environments within the Salinas Valley. Environmental sampling and WGS provide insight into the potential source and scope of contamination during outbreaks, which underscores the value of widespread microbiologic sampling of watersheds nationally.

In both outbreaks, leafy green exposure was reported in interviews, but brand information and purchase records needed for regulatory efforts were limited. Relying on available exposure data alone would not have solved these outbreaks. Obtaining complete exposure information is challenging for listeriosis outbreaks because patients are severely ill or may have died. In those instances, a surrogate might be interviewed who has limited knowledge of the patient’s food history. Patients themselves might have difficulty recalling food exposures and providing details like brands, purchase locations, and consumption dates when asked about foods eaten weeks or months before their illness. In the outbreaks described, packaged salad exposure was easily identified, but narrowing the source to a single firm was difficult because leafy green processors are often associated with multiple brands and can have similar packaging.

Given those limitations, routine and epidemiologically directed sampling efforts by GDA and MDARD were key to solving both outbreaks and provided laboratory evidence needed to justify regulatory inspections and product actions. Internal sampling, root cause analysis, and information sharing by firm X played a substantial role in outbreak A’s investigation. Firm X encourages industry partners to leverage forms of routine early surveillance, including sampling raw materials and using WGS to compare isolates in NCBI. They also emphasize the importance of investigating outside the production facility, such as field or farm investigations, in response to outbreaks (N. Dyenson, firm X, pers. comm., email, 2023 Apr 21).

During 2015–2024, a total of 8 US listeriosis outbreaks were linked to packaged salads (4 suspected and 4 confirmed) (*21*). During January 2015–May 2024, FDA classified (i.e., the final determination of violation and risk) recalls of ≈240 packaged salad products because of potential contamination with *L. monocytogenes* (*22*,*23*). Responsibility for food safety occurs along the entire farm-to-fork continuum of growing, harvesting, processing, distribution, and preparation. Research shows both romaine and iceberg lettuce can potentially internalize *L. monocytogenes* (*24*,*25*), in addition to the risk associated with colonization of *L. monocytogenes* during and after harvest. Although refrigerated storage supports the survival and growth of *L. monocytogenes* on both packaged and less processed leafy greens, packaged salads might be at higher risk for *L. monocytogenes* contamination than less processed leafy greens because they touch a higher number of surfaces and equipment during processing.

Outbreaks A and B caused 30 illnesses, 27 hospitalizations, and 4 deaths over 8 years. Those outbreaks provide further evidence of the ongoing risk for *Listeria* infections associated with packaged salad. Routine and epidemiologically directed sampling conducted by state partners solved these outbreaks, and the actions taken by both firms likely prevented additional illnesses and deaths. Actions taken by the firms provide examples of measures other producers can implement to identify and prevent contamination from harvest to packaging. Firms should consider WGS as a tool that can be leveraged to prevent illnesses. Sequencing pathogens identified within processing facilities can enhance internal microbial monitoring programs, enabling comparison of strains over time against known isolates or events in NCBI. Firms should consider applying a similar methodology to incoming raw material in processing facilities. Microbiological surveillance of incoming product could enable firms to identify potential risk before introducing pathogens into facilities. If foodborne pathogens are identified, firms should consider conducting internal root cause analysis both at the production facility and further upstream at harvesting sites. Leafy greens are part of a healthy diet, and more research should be done to identify improved production methods for preventing contamination.
